# Homocysteine thiolactone from translation proofreading is an endogenous ligand for cell-cell signaling by the receptor SdiA

**DOI:** 10.1128/mbio.01407-26

**Published:** 2026-07-16

**Authors:** Lorenzo Eugenio Leiva, Roberto A. Zúñiga, Gregory J. Phillips, Michael Ibba

**Affiliations:** 1 Schmid College of Science and Technology, Chapman University 6226, Orange, California, USA; 2 Department of Infectious Diseases, College of Veterinary Medicine, University of Georgia 1355, Athens, Georgia, USA; 3 Department of Microbiology, The Ohio State University 2647, Columbus, Ohio, USA; University of California, Berkeley, California, USA

**Keywords:** translation, methionine, tRNA, synthetase, proofreading

## Abstract

Several widespread mechanisms enable cellular communication and coordinate the behavior of bacterial populations. The modules responsible for cellular communication are often found in matching pairs: an enzyme that synthesizes a signal molecule and a specific receptor that decodes it. A notable exception is *Escherichia coli* SdiA, a homolog of known N-acyl-homoserine lactone (AHL) receptors, for which no corresponding AHL synthase exists. SdiA is an orphan receptor that enables cross-talk by sensing AHLs from other bacterial species. In this work, we investigated whether homocysteine thiolactone (HTL), an AHL-like molecule arising from a proofreading reaction of methionyl tRNA synthetase (MetRS) to correct misactivation of homocysteine, participates in *E. coli* communication through SdiA. A comprehensive analysis of MetRS variants producing different levels of HTL revealed more extended lag periods at lower HTL levels, which were restored by HTL supplementation. SdiA mutants abolished the HTL-induced regulation of the transition from the lag to the exponential phase. Growth phenotypes were HTL specific and were not induced by supplementation of other non-endogenous AHLs previously characterized as SdiA ligands. The gene expression of *gadY* and *rmf*, regulated by SdiA, was modulated by HTL supplementation. Through differential scanning fluorimetry, we show the SdiA-HTL interaction *in vitro*. Together, our observations describe HTL as an SdiA ligand that modulates cell-cell communication in bacteria.

**IMPORTANCE:**

Bacteria need to adapt to different environments and stress conditions, and quorum sensing (QS) is a widespread and well-established mechanism that enables cell-to-cell communication to coordinate these changes. The modules responsible for QS usually evolve in pairs: an enzyme that synthesizes the signal and a specific receptor that decodes it. A notable exception is *Escherichia coli* SdiA, a homolog of known N-acyl-homoserine lactone (AHL) receptors for which no corresponding AHL synthase exists. It has been hypothesized that SdiA is an orphan receptor that enables crosstalk by sensing AHLs from other bacterial species. We present data suggesting that homocysteine thiolactone, a ubiquitous molecule arising from homocysteine editing during translation proofreading, can mimic AHLs and participate in bacterial cell-cell communication via SdiA. The near-universal distribution of this proofreading mechanism suggests that homocysteine thiolactone provides a common link between protein synthesis and cell-cell communication in both homologous and heterogeneous microbial populations.

## INTRODUCTION

Bacteria need to adapt to different environments and stress conditions, and quorum sensing (QS) is a general cell-cell communication mechanism that synchronizes these changes when a specific critical density of a diffusible signal molecule is reached (1). The variety of known QS signal molecules has grown over the years. In gram-negative bacteria, autoinducers of type I (AI-I), based on acyl-homoserine lactones (AHLs), are the most commonly known class of QS signal molecules (2). They contain an N-acylated homoserine-lactone ring core and a 4–18 acyl chain (3). The features of aliphatic chains, such as length and possible modifications, give AI-Is a species-specific character and affect their stability, diffusion, and detection. For most of the bacteria able to produce AI-I, their synthesis is often catalyzed by LuxI-type synthases, which produce AHLs by deriving lactones from S-adenosyl methionine (SAM) and the acyl chain from intermediates of fatty acid metabolism (3).

The most common receptors for QS signals in gram-negative bacteria are LuxR-type proteins, which are cytoplasmic transcription factors. The formation of the LuxR-autoinducer complex promotes the stability of the receptor and its homodimerization and facilitates subsequent binding to *lux* boxes in the promoter region of target genes (4, 5). Despite the coevolution of the AHL synthase-receptor pair, based on amino acid variation in the binding pocket to give AHL binding specificity, close to 75% of the annotated LuxR homologs correspond to orphan transcriptional factors that are not accompanied by a LuxI synthase counterpart. These receptors are called LuxR-solo class proteins (6). One alternative explanation is that some of these LuxR-solo classes exhibit relaxed ligand-binding specificity, allowing recognition of many types of AI-I. For example, QscR is a well-studied LuxR-only receptor from *Pseudomonas aeruginosa*, whose activity can be modulated by C8-HSL, C10-HSL, 3-oxo-C10-HSL, C12-HSL, 3-oxo-C12-HSL, and C14-HSL (7, 8). Furthermore, there are examples of QS-related LuxR homologs that respond to endogenously produced non-AHL ligands; for instance, PluR and PauR respond to photopyrones and dialkylresorcinol, respectively (9, 10). SdiA is an *Escherichia coli* homolog of the LuxR receptor linked to bacterial virulence and tolerance, for which no signaling molecule produced by the same bacteria has been described (11–13). Instead, SdiA responds to ligands produced by other bacteria, a process termed “eavesdropping” (14, 15).

Protein synthesis depends on the aminoacyl tRNA synthetases, an essential family of enzymes that define the genetic code by translating the nucleotide alphabet into proteinogenic amino acids. The aminoacylation process consists of a two-step reaction: the first step involves the recognition and activation of the amino acid before its subsequent transfer to the cognate tRNA. To preserve the fidelity of the process, different mechanisms allow the editing of recognition errors resulting from physicochemical similarities between amino acids, such as phenylalanine and tyrosine, and branched amino acids (16). These errors can also involve non-proteogenic amino acids, such as D-amino acid enantiomers or intermediates in amino acid biosynthesis. An example of the latter is homocysteine (Hcy), a sulfonated amino acid intermediate in the biosynthesis of methionine (Met) from oxaloacetate in the TCA cycle (17). Although Hcy lacks the final methylation in the R group, methionyl tRNA synthetase (MetRS) can recognize and activate this amino acid. However, the hydrophobic environment of the amino acid binding pocket promotes a highly efficient cyclization reaction between the reactive sulfhydryl group of Hcy and the carboxyl carbon, releasing AMP and producing homocysteine thiolactone (HTL) (18). HTL is a stable molecule at acidic and neutral pH values, is highly diffusible at physiological pH, and has significant similarity to the lactone ring of AHLs (19, 20).

HTL has been considered a so-called carbon-leak point for many years because no biological reaction in bacteria has been described that reintegrates HTL into bacterial metabolism. Deleterious effects of HTL accumulation have been described due to the reactivity of the sulfur group and its ability to modify exposed lysines in eukaryotic proteins, but not bacteria (21). In this work, we describe how *E. coli* produces HTL mainly via proofreading by MetRS and how its subsequent accumulation triggers exponential growth without affecting the duplication time. This suggests that HTL has the potential to act as a signaling molecule mimicking AHLs. Additionally, we show that this phenotype is dependent on the presence of the LuxR-solo receptor SdiA. The implications of HTL accumulation and detection in the bacterial population for regulating the lag period of growth and for bacterial antibiotic tolerance are also discussed.

## RESULTS

### Changes in the lag period in response to HTL levels

**Fig 1:**
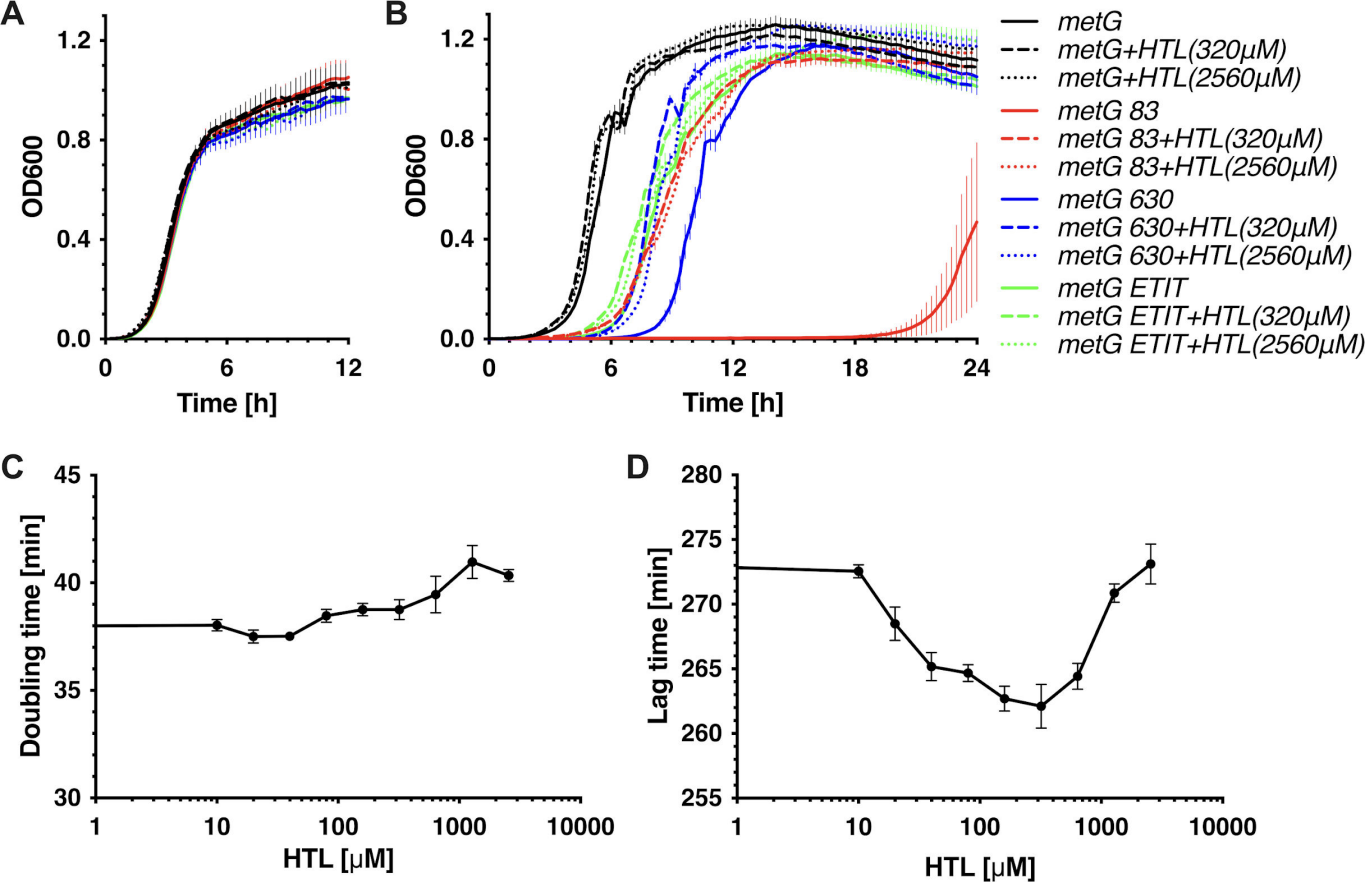
*metG* mutations impact the lag period of growth curves. All strains were grown in LB (**A**) or M9 medium (**B**). M9 medium was supplemented with 40 µg/mL of each amino acid except Met. The OD at 600 nm (OD600) of *E. coli* strains *metG* (wild type), *metG83*, *metG630*, and *metG ETIT* was monitored in a microplate reader at 37°C with constant shaking. Error bars represent standard deviations of biological triplicate. Strains were supplemented with 320 µM or 2,560 µM HTL when indicated. The doubling time (**C**) and lag time (**D**) were determined for the *metG* strain grown at increasing concentrations of HTL.

*E. coli metG* is an essential gene that encodes MetRS, an enzyme responsible for the aminoacylation of both tRNA^fmet^ (tRNA_i_) for translation initiation and tRNA^met^ for elongation. To initiate our studies, we selected a set of *metG* mutants that our previous research showed were deficient in MetRS proofreading activity (22). These strains contain mutations that alter amino acids near the catalytic site of MetRS (*metG83*), delete four amino acids near the anticodon-binding domain (*metETIT*), and truncate the last 47 amino acids that form part of the tRNA binding domain, recapitulating a mutant first isolated in *S. Typhimurium* (*metG630*). When analyzing cell growth, all *metG* mutants showed the same growth patterns as their parental strain in lysogeny broth (LB) medium (Fig. 1A). Because LB contains about ∼5.9 mM Met (23), any defect in MetRS proofreading activity could be masked by an excess of the cognate amino acid. Repeating the same analysis in a medium without Met, the strains showed greater differences in the lag period, but not in the doubling time, during the exponential phase of growth (Fig. 1B and C).

MetRS, like the isoleucine, leucine, and valine aminoacyl-tRNA synthetases, can bind and activate Hcy. However, due to the proofreading activity of these enzymes, Hcy adenylate is rapidly removed from the amino acid-binding pocket via a cyclization reaction that releases HTL. To explore whether changes in HTL production rate are related to the differences observed in the lag period, the medium without Met was supplemented with HTL. A notable reduction in the lag period was observed in the *metG83* and *metG630 strains*, while a slight effect was observed in the wild type (WT) and *metGETIT* strains (Fig. 1B). The reduction in the lag period of the WT gradually decreased to a minimum upon the addition of 320 μM HTL, after which the lag period began to increase (Fig. 1D), probably due to the deleterious effects that have been attributed to elevated levels of HTL (24, 25). Higher levels of HTL in *metG83* and *metG630* did not lead to a significant increase in the lag period, suggesting that after a threshold concentration, there is no dose-response effect in decreasing the lag period (Fig. 1B). Within the same HTL titration range, differences of less than one minute in doubling time were observed up to 320 µM, with an increase once the concentration exceeded 1 mM HTL (Fig. 1C). A previous report (22) found that all *metG* mutants analyzed in this study exhibit a slower rate of protein synthesis, without a significant effect on growth. Using fluorescent reporters, we corroborated a slight trend toward slower protein synthesis in our culture conditions in M9 medium, a difference that is minimized when the medium is supplemented with Met (Fig. S1). However, none of these differences in protein synthesis correlates with the magnitude of the differences in lag phase induced by HTL. From these results, we can infer that mutations in *metG* affect MetRS’s ability to produce and accumulate a critical concentration of HTL.

### Long lag periods correlate with low HTL concentrations

**Fig 2:**
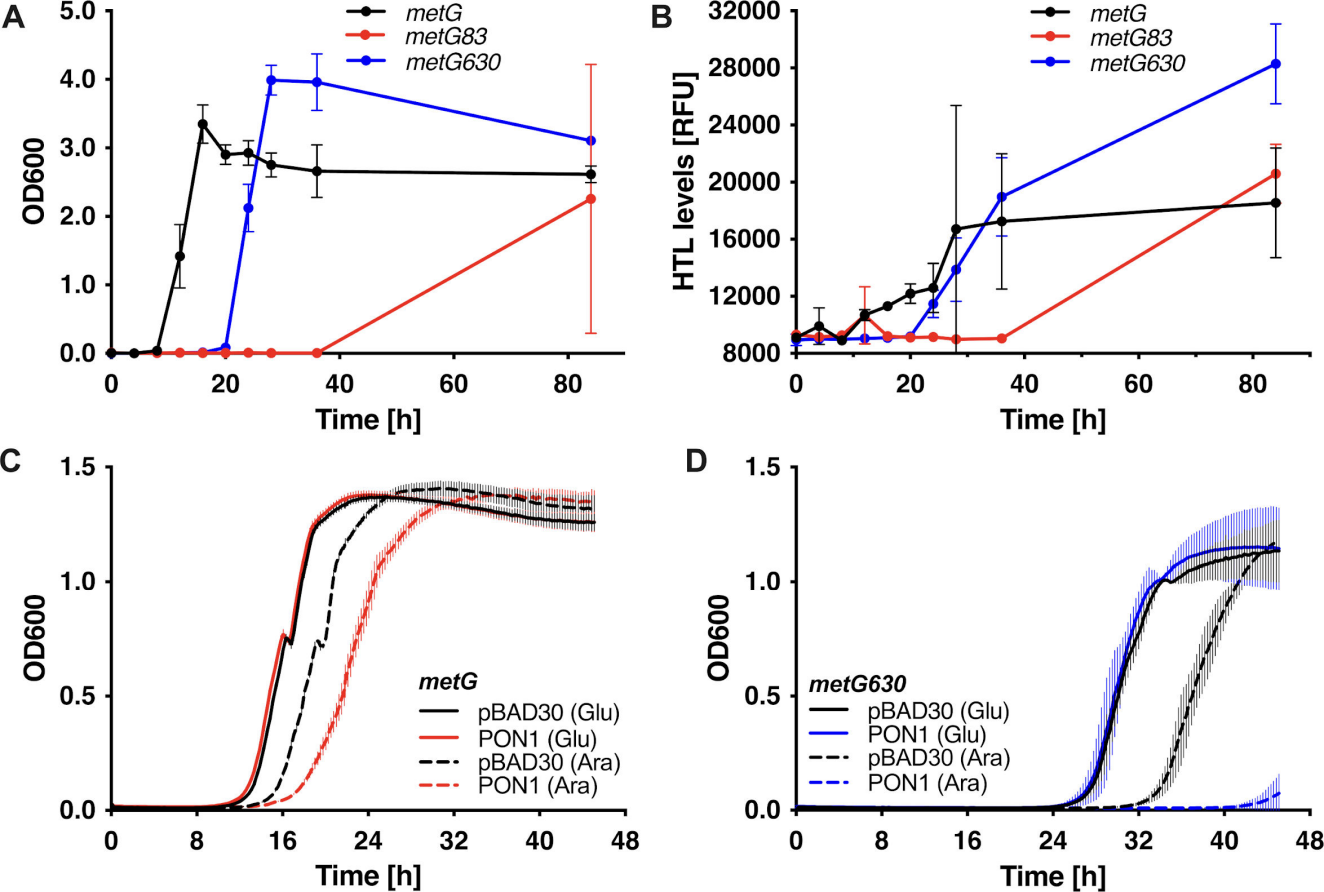
Lag period in response to HTL levels. (**A**) *metG* (wild type), *metG83*, and *metG630* growth curves in M9 medium without amino acid supplementation (pre-inocula and cultures). Cultures were carried out in test tubes at 37°C with constant shaking. (**B**) Aliquots of the culture supernatants in Figure A were mixed with an orthophthalaldehyde (OPA) solution to produce a fluorogenic derivative of HTL and measure its levels on a TECAN plate fluorimeter. Error bars represent the standard deviation between biological replicates. PON1, a hydrolase that catalyzes the degradation of a wide range of lactones, was cloned into plasmid pBAD30 and transformed into *metG* (wild type) (**C**) and *metG630* (**D**). Strains were grown in M9 medium supplemented with glucose (Glu) or arabinose (Ara) to repress or induce PON1 expression, respectively.

To determine whether strains *metG83* and *metG630* respond to exogenous HTL supplementation due to a deficiency in its synthesis, HTL levels were measured over time (*metGETIT* was excluded from this and subsequent analyses due to the small effect size of HTL supplementation). HTL monitoring was based upon two considerations: (i) because HTL can quickly diffuse through the membrane and no differences in intracellular levels have been observed in Met biosynthesis mutants that produce higher levels of Hcy and HTL, their levels were monitored from the supernatant (21, 26). (ii) Because HTL synthesis can be reduced due to amino acid supplementation (27), we used M9 minimal medium where no amino acids were added in cultures or preinocula. Although this strongly affected the extension of the growth curves, the same differences persisted in the lag periods between the WT, *metG83*, and *metG630* strains (Fig. 2A). To determine HTL levels in the supernatant, we established a fluorometric plate assay based on the reaction between HTL and *ortho-*phthalaldehyde (OPA) (26, 28). We observed different dynamics of HTL levels among the strains, with the most rapid accumulation in the supernatant observed in the WT, followed by *metG630* and *metG83* (Fig. 2B). This is consistent with more extended lag periods in the growth curve being correlated with lower levels of HTL synthesized by the cell.

To further corroborate that the effect on culture growth is not an artifact of mutating *metG*, we attempted to reduce HTL levels independently of MetRS proofreading activity. To achieve this goal, we induced the expression of a variant of paraoxonase 1 (PON1), a calcium-dependent hydrolase that catalyzes the degradation of a wide range of lactones. Because the origin of this enzyme is mammalian and shows low solubility in *E. coli*, we used a recombinant variant evolved *in vitro* for bacterial expression (29, 30). The toxic effect of overproducing PON1 was minimized by cloning it into a low-copy-number plasmid inducible by arabinose supplementation. To investigate the lag period in response to PON1 induction, we compared WT and *metG630* strains transformed with either the parental plasmid pBAD30 or the pPON1 plasmid (Fig. 2C and D). Both plasmids induced the same growth curve dynamics in WT and *metG630* when the medium was supplemented with glucose to repress expression. However, when cultures were supplemented with arabinose, pPON1-transformed strains showed a more extended lag period before exponential growth, supporting the hypothesis that HTL levels regulate the onset of exponential growth (Fig. 2C and D). The more efficient growth observed in the presence of glucose can be explained by its immediate use in glycolysis, whereas arabinose enters metabolism via the pentose phosphate pathway.

### Response to HTL and pH in the medium

**Fig 3:**
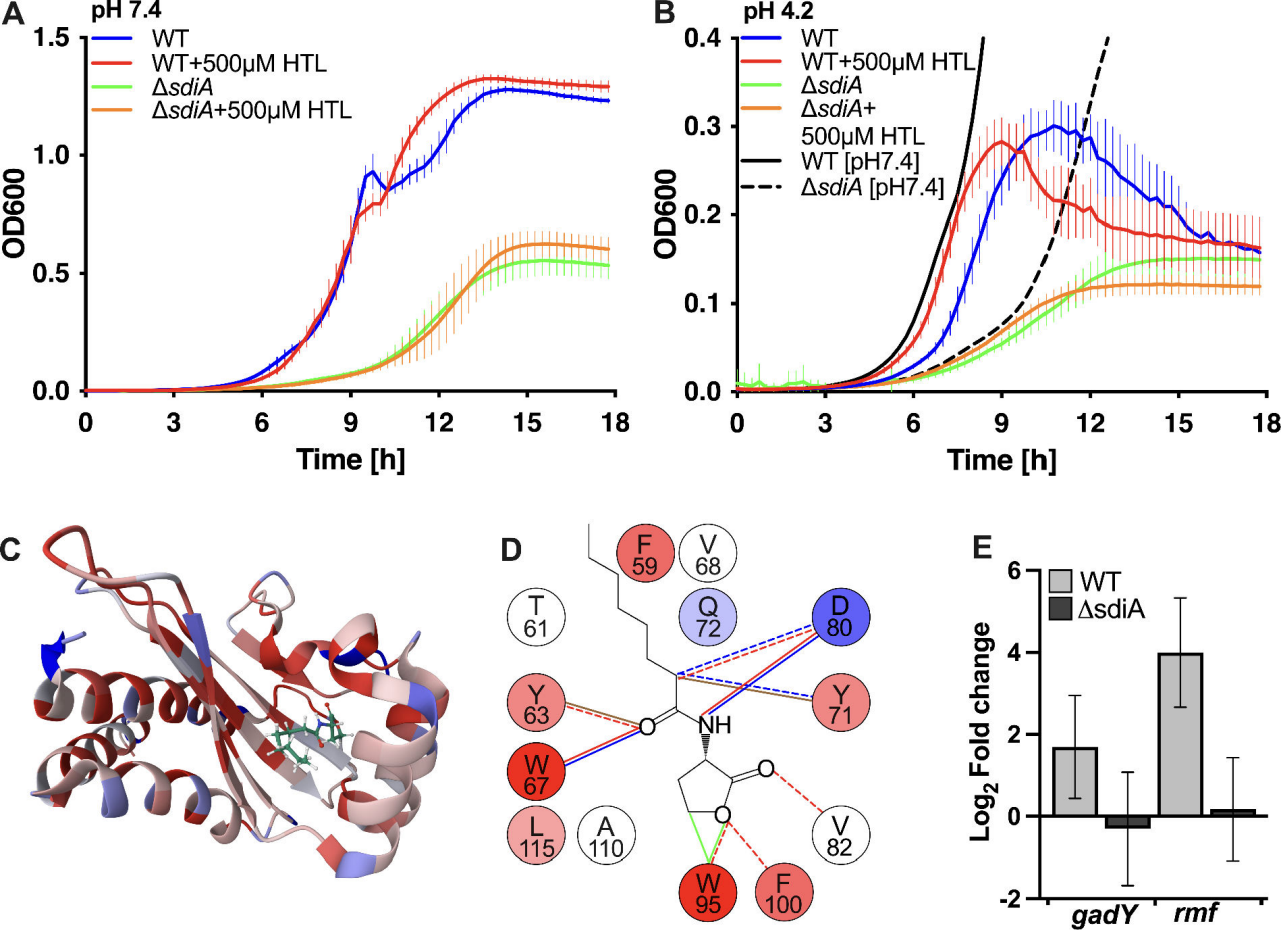
Determination of the role of SdiA in HTL response. *metG* (wild-type) and *metGΔsdiA* (ΔsdiA) were cultured in M9 medium at pH 7.4 (**A**) and pH 4.2 (B) and supplemented with 500 µM HTL when indicated. The OD at 600 nm (OD600) was monitored in a microplate reader at 37°C with constant shaking. Error bars represent standard deviations of biological triplicate. (**C**) The protein structure of a complex between the ligand-binding domain of SdiA and the autoinducer N-octanoyl-L-homoserine lactone (C8-HSL) was calculated using NMR data. The colors represent the hydrophobicity scale: hydrophobic (red) and hydrophilic (blue). (**D**) Description of the autoinducer binding pocket and its interactions: hydrogen bond (blue lines), polar (red lines), van der Waals (green lines), and hydrophobic (brown lines). Weak interactions are presented with a dashed line. (**E**) Fold change of *gadY* and *rnf* transcripts between cultures in M9 and M9 supplemented with HTL 320 µM for 30 min, for WT and Δ*sdiA* strains.

The finding that HTL can regulate the duration of the lag period raises the question of whether intracellular HTL concentrations are sufficient to trigger exponential growth or whether HTL levels coordinate the transition to exponential growth based on extracellular HTL concentration. Before exploring this last alternative, we must consider the properties of the molecule and the changes in the medium during bacterial growth. HTL is a small molecule with a pKa of 6.67, which makes it primarily uncharged at neutral pH, allowing it to diffuse across membranes (31, 32). HTL has been reported to be very stable under acidic conditions over various temperatures (33). On the contrary, alkaline conditions can induce its degradation by opening the thiolactone ring and reacting with other HTL molecules, forming 2,5-diketopiperazine Hcy (34). This becomes relevant when the pH of the media fluctuates depending on the carbon source and oxygenation levels, leading to alkalinization due to amino acid metabolism or acidification due to sugar fermentation (35, 36). Next, to explore whether fluctuations in extracellular HTL levels are more relevant than intracellular HTL levels in triggering exponential growth of the biomass, we analyzed the response to HTL supplementation at different pHs in the WT strain. When we examined the growth curves at pH 7.4, where 84.3% (269.8 µM) of the total supplemented HTL was in the neutral form (based on the Henderson–Hasselbalch equation), the lag period was not reduced by HTL supplementation (Fig. 3A). However, at a pH of 4.2, the WT strain exhibited a prolongation of approximately 1 hour in the lag phase, which was restored following supplementation with 500 µM HTL (of which only 0.34% [1.1 µM] was neutral, according to the Henderson–Hasselbalch equation). Together, these observations suggest that under nutrient-limited growth conditions, the intracellular levels of HTL produced as by-products of Met metabolism are insufficient to trigger exponential growth.

### SdiA is required to detect HTL

Although our results showed that HTL is a molecule involved in modulating growth dynamics, to support that it is part of a communication mechanism, it is necessary to elucidate how HTL is detected. HTL shares a similar structure with the homoserine-lactone ring of AI-I, raising the possibility of crosstalk with QS receptors. SdiA, a homolog of the AI-I receptor LuxR, is present in *E. coli* and has been associated with cell division, antibiotic resistance, virulence in pathogenic strains, biofilm formation, and acid resistance, among other phenotypes (11, 13). While no ligand for SdiA synthesized by *E. coli* has been described to date, its role in detecting AHLs derived from other bacterial species is well documented (14). Consequently, SdiA is a QS receptor that modulates *E. coli* physiology in response to population changes in different environmental species (14). Structural studies have analyzed the interaction between SdiA and N-octanoyl-L-homoserine lactone (C8-HSL), showing that many of the most conserved amino acids of the binding pocket interact with the ketone, the amino, and the β carbon of the lactone, all of which are also present in HTL (Fig. 3C and D) (5).

To study the role played by SdiA in regulating the lag period dependent on HTL levels, we analyzed the response to HTL supplementation in a ∆*sdiA* mutant (∆*sdiA::kan*). Because this *sdiA* mutation itself is sufficient to cause a defect in the growth of *E. coli*, to allow analysis in microplates in a time window no greater than 24 h, the effects of neither ∆*sdiA*, *metG83*, nor ∆*sdiA*, *metG630* double mutants were investigated. While WT and ∆*sdiA E. coli* showed no response to HTL supplementation at pH 7.4, at acidic pH, where the modulation of the lag period is better observed in WT, the response to HTL addition disappeared in the ∆*sdiA* mutant (Fig. 3A and B). This result supports the idea that HTL is a ligand for SdiA.

Several reports that have analyzed the function of SdiA have described targets of this transcription factor, such as *gadY*, which is involved in acidic tolerance, and *rmf*, which encodes a ribosomal modulation factor that coordinates the transition to the stationary phase (11, 37). Because we expect the SdiA transcription factor to be affected by HTL levels, we compared the expression levels of *gadY* and *rfm* in cultures incubated for 30 min in M9 medium with or without HTL (Fig. 3E). These results showed increases in the expression of both genes of ~4- and ~20-fold compared to the culture without HTL supplementation. This effect of HTL on *gadY* and *rfm* expression is absent in the ∆*sdiA* mutant, showing that HTL-induced expression changes depend on SdiA (Fig. 3E).

**Fig 4:**
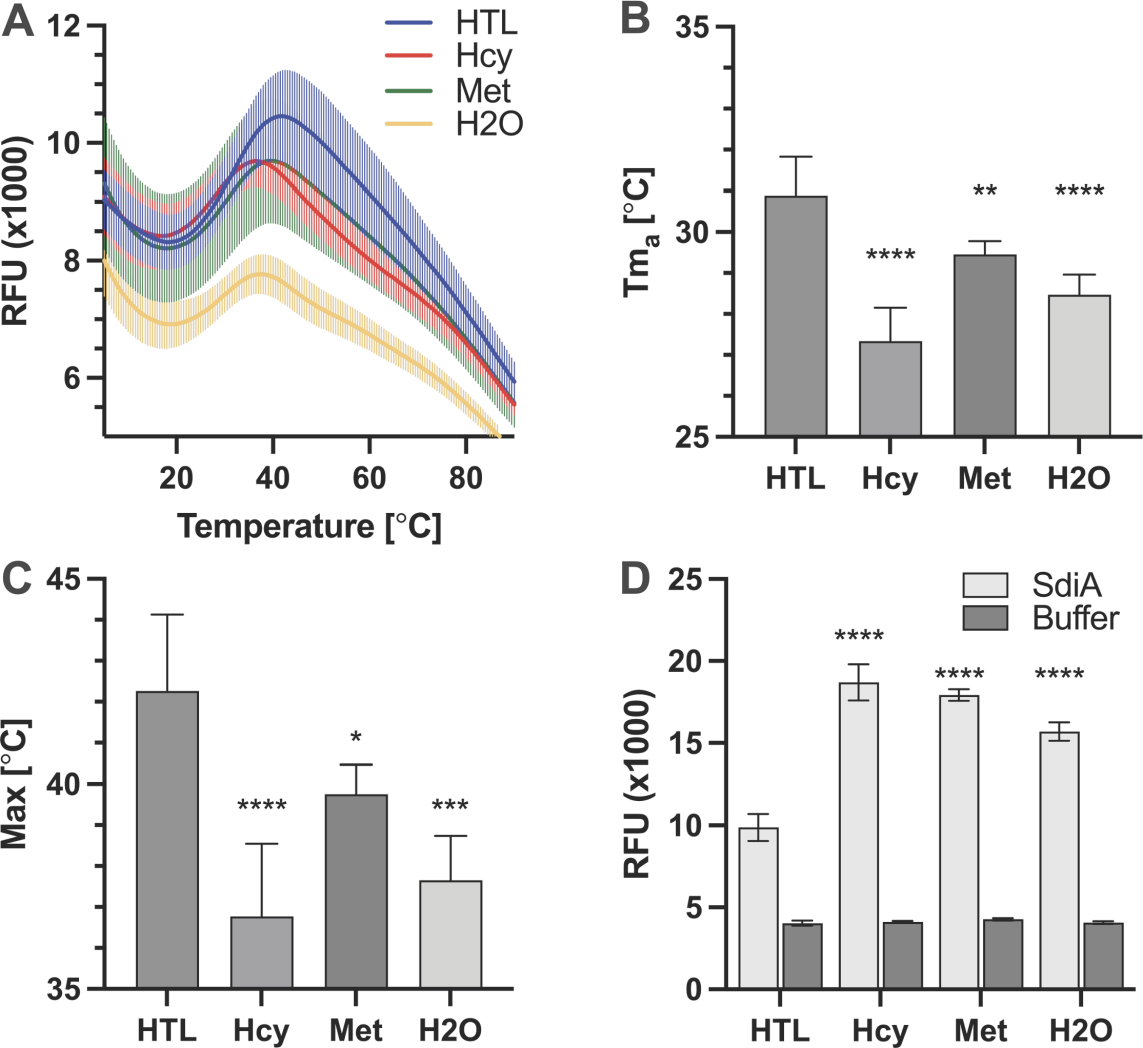
HTL stabilizes SdiA and quenches fluorophore binding to hydrophobic regions. Measurements of SdiA stability in response to HTL, Hcy, and Met were performed using differential scanning fluorimetry. (**A**) SdiA unfolding curve in the presence of SYPRO Orange in a 1:2 ratio. Potential ligands were used at equimolar concentrations (5.4 mM). The apparent melting temperature (Tm*ₐ*) (**B**) and fluorescence maximum (Max) (**C**) were determined from panel **A**. (**D**) Measurement of fluorophore quenching (at a ratio of 1:1.7) at 4°C. The background without protein (buffer) is presented in gray. ****P ≤ 0.0001, ***P ≤ 0.001, **P ≤ 0.01, and *P < 0.05, one-way analysis of variance with Dunnett versus HTL.

To support our model that the observed response to HTL is a consequence of direct interaction with SdiA, we assessed their interaction by using differential scanning fluorimetry to monitor changes in the thermal stability of SdiA in the presence of the ligand (38, 39). Protein stability depends on physical parameters (temperature, salinity, pH, detergents, etc.) and biophysical factors, such as protein-ligand interactions (38, 40). We analyzed SdiA unfolding by measuring SYPRO Orange fluorescence intensity, which depends on the dye’s interaction with the hydrophobic regions of SdiA exposed during thermal denaturation (Fig. 4A). The vehicle for all ligands was water, which served as the baseline for the unfolding profile, the apparent melting temperature (Tm_*a*_), the temperature of fluorescence maximum (Max), and the fluorophore quenching. The presence of HTL shifted the unfolding profile to higher temperatures (Fig. 4A) and increased the Tm_*a*_ and the temperature that maximized fluorescence for the unfolded protein (Max; Fig. 4B and C). This change in the Tm suggests that the interaction with HTL stabilizes SdiA. This stabilization effect on SdiA was not observed with Hcy, the cyclization reaction’s precursor amino acid substrate, which showed values similar to those of the vehicle (Fig. 4A, B and C). Because the effect of HTL supplementation in a Met-rich medium was not observed (Fig. 1A), the interaction of SdiA with Met was also evaluated. Although there is a slight increase in melting temperature with the addition of Met, it is less than that observed for HTL. Given the abundance of hydrophobic amino acids in the ligand-binding domain (Fig. 3C), the temperature change with Met could result from its greater hydrophobicity. The binding pocket of SdiA provides a highly hydrophobic environment for the ligand, which is also occupied by the fluorophore during the assay due to its affinity for these hydrophobic regions. When we measured fluorescence immediately after adding the dye to the protein-ligand complex, we observed that only HTL could quench the fluorophore, suggesting that SYPRO Orange rapidly displaces Hcy and Met. At the same time, HTL remains in the binding pocket due to its higher affinity (Fig. 4D). Together, these results strongly suggest that HTL plays a regulatory role with SdiA as its receptor.

It is important to note that the results presented in this study reveal the different factors involved in the phenotype associated with this signaling process. However, the fine-tuning required for its activation is still unclear, making it difficult to replicate the same response in other strains. To investigate this aspect, we analyzed the effect of HTL on the K12 MG1655 strain and its *sdiA* mutant (∆*sdiA::frt*). While HTL supplementation induced exponential growth in this new strain, the impact of *sdiA* was not observed as in BW30270 (Fig. S2). This unexpected inconsistency prompted us to investigate the differences between the two strains, and sequencing revealed that the BW30270 strain has a deletion in a locus containing 15 genes involved in bacterial motility and its regulation (Fig. S2). Some of these genes are part of the flagellar motor, while others regulate chemotaxis. Due to the high energy cost of building and operating the flagellar apparatus (41), the mutation in BW30270 results in a different metabolic state between the two strains. Furthermore, since BW30270 lacks *che* genes, SAM utilization during chemotaxis will be altered, affecting the use of Met and Hcy and HTL production. All of this makes a direct comparison of phenotypes between BW30270 and MG1655 impossible.

### Specificity of response to HTL

**Fig 5:**
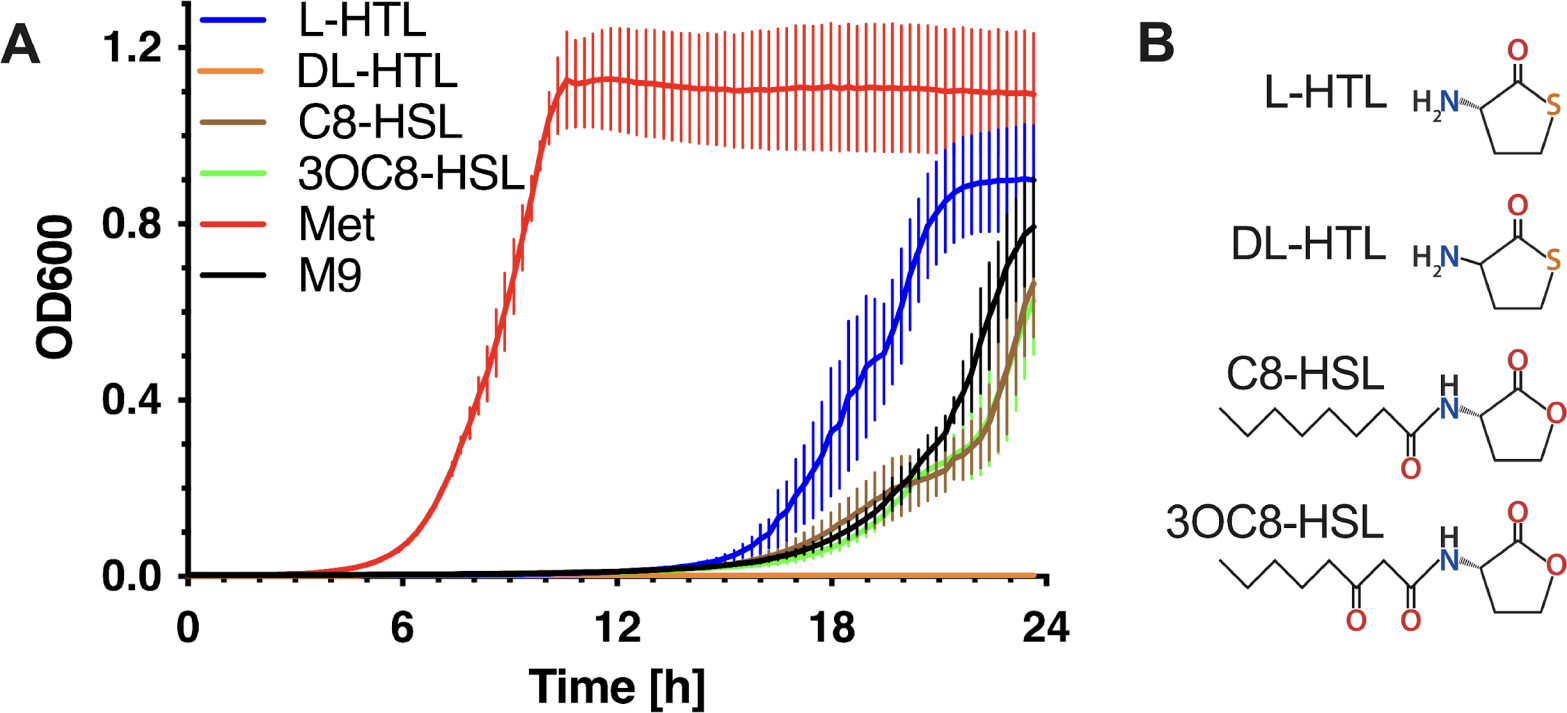
Effect of HTL analogs on the regulation of lag period. (**A**) *metG83* was cultured in M9 medium (without any additional amino acids) and supplemented with 320 µM of L-homocysteine thiolactone (L-HTL), methionine (Met), N-octanoyl-L-homoserine lactone (C8-HSL), N-(3-oxooctanoyl)-L-homoserine lactone (3OC8-HSL), and the racemic mixture of D-HTL and L-HTL (DL-HTL), when indicated. The OD at 600 nm (OD600) was monitored in a microplate reader at 37°C with constant shaking. Error bars represent standard deviations of biological triplicate. (**B**) HTL homologs added to the culture.

Previous studies have examined the ability of SdiA to eavesdrop on exogenous ligands through the use of sensitive transcriptional fusions or the crystallization of the SdiA-AHL complex, employing various autoinducers such as C8-HSL and 3OC8-HSL derived from *Burkholderia* spp., *Pseudomonas* spp., and *Agrobacterium* spp. (5, 42–44). Although gene expression was detected and the SdiA-AHLs complex was capable of co-crystallizing, no marked phenotypes were associated with this fact. To analyze the selectivity of the phenotype described in this work with respect to HTL, via its interaction with SdiA, we compared the effect on the lag phase during the growth of *metG83* following supplementation with equimolar concentrations of a set of HTL analogs (Fig. 5). With Met supplementation, we observed a drastic reduction in the lag period, consistent with the rapid growth of the metG83 mutant previously observed in LB medium (Fig. 1 and 5). Upon analyzing the effect of exogenous AHLs on our *metG83* strain, we observed that the lag periods following C8-HSL and 3OC8-HSL supplementation are longer than those for bacteria supplemented with HTL and are closer to those grown in M9 medium. A molecular docking analysis between the ligand-binding domain cavity of SdiA and a set of potential ligands, including L-HTL and N-acyl HSLs with varying aliphatic chain lengths and modifications, showed that L-HTL had the highest ligand efficiency, indicating a more favorable binding (Table 1). This prediction is consistent with the triggering of bacterial growth in the presence of L-HTL and the absence of a response in the presence of other ligands such as C8-HSL and 3OC8-HSL.

**TABLE 1:** Prediction of the interaction between the ligand-binding domain cavity of SdiA and potential ligands^*a*^

Ligand acronym	Ligand name	Formula	Vina score (kcal/mol)	Ligand efficiency (kcal/mol per heavy atom)
L-HTL	L-homocysteine thiolactone	C_4_H_7_NOS	−4.3	0.61
C4-HSL	N-butyryl-L-homoserine lactone	C_8_H_13_NO_3_	−6.7	0.56
C6-HSL	N-hexanoyl-L-homoserine lactone	C_10_H_17_NO_3_	−7.4	0.53
3OC6-HSL	N-(3-oxohexanoyl)-L-homoserine lactone	C_10_H_15_NO_4_	−7.4	0.49
C8-HSL	N-octanoyl-L-homoserine lactone	C_12_H_21_NO_3_	−8.1	0.51
3OC8-HSL	N-(3-oxooctanoyl)-DL-homoserine lactone	C_12_H_19_NO_4_	−9.4	0.55
C10-HSL	N-decanoyl-L-homoserine lactone	C_14_H_25_NO_3_	−8.1	0.45
C12-HSL	N-dodecanoyl- L-homoserine lactone	C_16_H_29_NO_3_	−7.4	0.37

^*a*^ The table describe the prediction of ligand efficiency (−∆G/number of heavy atoms) between SdiA (AF-P07026-F1-model-v4) and the ligands L-HTL, C4-HSL, C6-HSL, 3OC6-HSL, C8-HSL, 3OC8-HSL, C10-HSL, and C12-HSL.

The source of HTL in our model is L-Hcy, which is converted to L-HTL during MetRS editing. However, the DL-HTL mixture was described as an anti-quorum-sensing molecule synthesized by coral symbiotic bacteria (45). We found that DL-HTL is insufficient to obtain the critical concentration of L-HTL to reduce the lag period and inhibit growth, suggesting that D-HTL is the enantiomer responsible for the previously reported inhibitory effects of HTL.

## DISCUSSION

### Homocysteine thiolactone is a determinant of lag-to-log phase transition during growth

**Fig 6:**
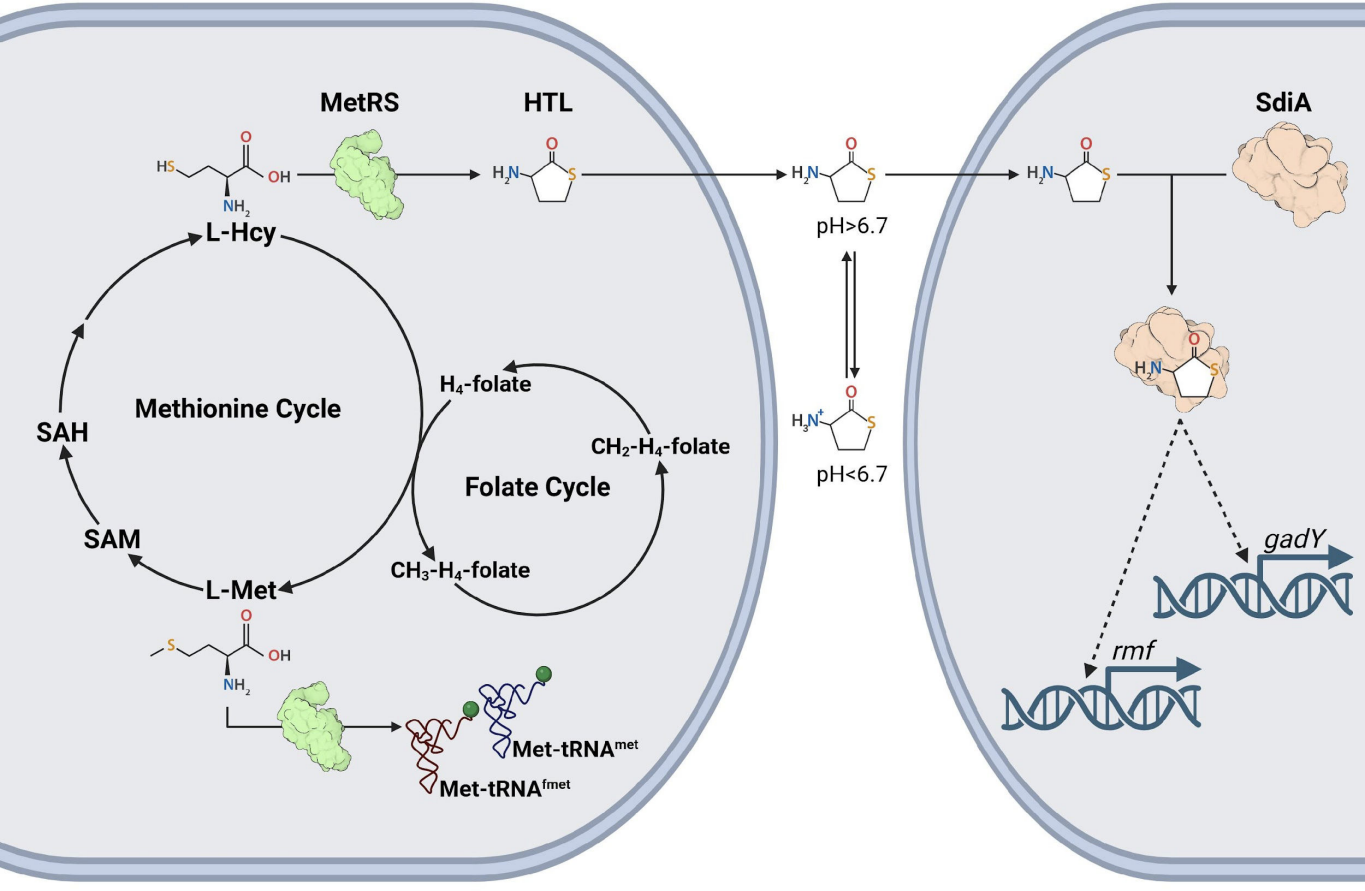
HTL synthesis in response to the balance between L-Hcy and L-Met. The conversion of L-Hcy to L-Met will depend on H4-folate levels. This new L-Met will be used for both translation and SAM synthesis. Consequently, the balance of L-Hcy and L-Met will depend on the state of the folate cycle, the level of protein synthesis, and the regeneration of L-Hcy in the methionine cycle. Any change in these central processes can induce the accumulation of L-Hcy, its subsequent misactivation by MetRS, and the synthesis of HTL. HTL can quickly diffuse at physiological pH owing to its size and neutrality. After reaching a critical concentration, which depends on extracellular pH, HTL enters cells, activates SdiA, and induces the expression of genes such as *gadY* and *rnf*.

Here, we describe a previously unrecognized endogenous ligand for SdiA, a LuxR-solo class protein, which senses HTL levels to coordinate *E. coli* growth. Bacteria such as *E. coli* encode the complete Met biosynthesis pathway, which involves the synthesis of Hcy as a precursor. Due to the chemical similarities between Hcy and Met, MetRS recognizes and activates Hcy, forming Hcy adenylate. The proofreading activity of MetRS then catalyzes the cyclization of Hcy to avoid translation errors, generating HTL as a byproduct (Fig. 6). Although the accumulation of this molecule was consistently associated with protein homocysteinylation damage (46), we show that HTL accumulation is necessary to modulate the transition between the lag and exponential growth phases (Fig. 1B). This new finding depended mainly on using *metG* mutants, initially identified in experiments designed to select strains resistant or highly tolerant to antibiotics or amino acid analogs. Regulation of the lag period, particularly its extension, is one of the mechanisms described that can induce stress tolerance and is vital for antibiotic tolerance (47–49). Given this, the lengthening of the lag period for the *metG* mutants provides a plausible explanation for the high antibiotic tolerance, i.e., persistence, observed for the strains (22).

The extended lag phases observed in the *metG* mutant strains could primarily be correlated with reduced HTL levels rather than a general reduction in protein synthesis rates, as no significant changes in translation activity were observed compared to the parental strain. Also, in an amino acid-limited minimal medium, HTL supplementation reduced the lag time of the growth curves without significantly modifying the doubling time (Fig. 1). The central role of HTL was further corroborated in a WT strain, where the expression of PON1, an enzyme that hydrolyzes the thiolactone ring to form Hcy, prevented the accumulation of HTL. The same effect was observed when the HTL permeability was reduced by lowering the culture pH, allowing the signal molecule to gain charge and preventing detection of HTL accumulation in the extracellular medium (Fig. 3B).

Here, we explored the dynamics and effects of HTL accumulation in *metG* mutants, but its effects may not be restricted solely to the early stages of growth. Previous work has shown that HTL can elevate *rpoS* levels during the stationary phase, although the mechanism underlying this regulation remains unclear (50). In addition, we reported that the *metG630* mutant accumulates more HTL than the parental strain over long periods, although we cannot rule out that it is a direct consequence of deregulation of the fine-tuning of Met biosynthesis/consumption due to changes in the interaction between the truncated MetRS in *metG630*, Hcy, and Met. In the context of a monoculture, these higher levels could affect gene expression during the stationary phase, although we need to consider that *sdiA* is also regulated during that phase, for example, through post-translational regulation by CsrA (51). However, in the context of a bacterial community, changes in the HTL plateau could affect the behavior of the entire population.

### Homocysteine thiolactone as a signal of amino acid homeostasis

Previous reports have shown how the level of HTL is affected by fluctuations in the balance of intracellular Hcy and Met concentrations. There, higher levels of HTL were detected rather than the higher levels of Hcy that might be expected in *metE* mutants, one of the two genes involved in the final methylation of Hcy in Met synthesis (27). This suggests that MetRS proofreading prevents the accumulation of excess Hcy that might otherwise disrupt branched-chain amino acid biosynthesis, thereby maintaining a balance between Hcy and Met and producing a molecule that reflects their ratio. It is essential to consider that fluctuations in other central metabolism cycles will also affect this balance. For example, cobalamin deficiency has been associated with Hcy accumulation in various organisms due to alterations in the folate cycle (52). When the latter is altered, tRNA_i_ formylation is reduced, affecting Met use in translation and contributing to an unbalanced Hcy/Met ratio (53). The Hcy/Met balance can also be affected by the methionine cycle, in which Met serves as a substrate for SAM synthesis, and SAH recycling produces more Hcy (Fig. 6). These observations suggest that HTL acts as a signal molecule whose concentration is determined by the state of different areas of central metabolism and that it must reach a threshold concentration to promote the transition from log to exponential growth.

### Homocysteine thiolactone signaling via SdiA

The core of the AI-I-based sensing mechanism consists of a LuxI-type synthase and a LuxR-type receptor, which catalyze the acylation and lactonization reaction between SAM and an acyl carrier protein, producing an AHL and recognizing and triggering a response to a threshold concentration of ligand, respectively (54). However, *E. coli* and many other bacteria lacking AHL synthase have orphan LuxR-type receptors.

The high conservation of residues in the ligand and DNA-binding domains, and their role in regulating several phenotypes, indicate that these receptors are not pseudogenes; these receptors recognize AHLs and other signals produced by different bacteria for interspecies communication (55, 56). Our genetic and biochemical analysis indicates that the presence of SdiA is also necessary to respond to HTL in the regulation of lag time (Fig. 3B) and that, as a consequence of their interaction (Fig. 4), SdiA exerts its role as a transcriptional factor, favoring the expression of genes such as *gadY* and *rmf* (Fig. 3E). Rather than contradicting the idea that *E. coli* uses SdiA to eavesdrop on its neighbors, our data show that SdiA can also sense endogenous HTL produced by *E. coli* to regulate the transition from the lag phase to the exponential phase of growth. This phenotype was not observed for other ligands, such as C8-HSL and 3OC8-HSL (Fig. 5A).

Due to the potential use of AHLs for bacterial control in both biomedical and biotechnological contexts, some studies have evaluated libraries of AHL homologs for their ability to activate QS receptors with known ligands and orphan receptors. These works have revealed that LuxR-type receptors can recognize molecules in which the lactone ring is replaced by a thiolactone ring, suggesting that the interaction between residues W95 and F100 (Fig. 3D), conserved in SdiA and other LuxR homologs, is with the sulfur of thiolactones (57, 58). Structural studies have shown that SdiA has selectivity for short-chain AHLs because the ligand binding pocket cannot accommodate aliphatic chains longer than eight carbons, a requirement met by HTL (59). Since intracellular HTL arises from the L-Hcy editing reaction, all HTL produced by MetRS corresponds to the L-HTL enantiomer, which our data suggest is stereospecific for SdiA activation. This specificity allows us to reinterpret some of the experiments in which, in the search for ligands for LuxR-like receptors from *E. coli* and *S. enterica*, the activation of SdiA was analyzed using DL-HTL, for which no effect was observed (60). Other molecules unrelated to the general structure of AHLs have been investigated as ligands of LuxR homologs, such as 1-octanoyl-rac-glycerol (monocaprylin). This molecule has been proposed as an endogenous ligand for SdiA because it was copurified with monocaprylin, which occupied the ligand-binding pocket (61). Because this molecule lacks a lactonic ring structure, part of the previously described interactions between SdiA and C8-HSL disappear. The authors proposed that these missing interactions are compensated for by glycerol molecules that fill the gaps in the binding pocket. On the other hand, this molecule has been shown to interact with the membrane, and its accumulation exhibits antimicrobial properties by destabilizing the membrane (62). Considering that no biological effect on SdiA has been observed in the presence of 1-octanoyl-rac-glycerol, it is not clear that it is a functional ligand of SdiA.

### Homocysteine thiolactone provides a general signal for cell signaling

Since Hcy and MetRS are found in all bacteria, the accumulation of HTL in response to changes in amino acid homeostasis is expected to be a common occurrence. This suggests that a mechanism similar to that observed in *E. coli* could occur in bacteria lacking AI-I synthetases, where virulence has been associated with SdiA activity, such as in *Salmonella* spp., *Klebsiella* spp., and *Shigella* spp., among others (12, 13). An exciting question arises when we integrate this new concept into bacteria that actively produce autoinducers for QS. Recently, HTL was proposed as an anti-QS molecule because it inhibits QS in the bioreporter *Chromobacterium violaceum* and *P. aeruginosa* (45). Although it is not clear whether the observed anti-QS effects were due to the use of the racemic mixture DL-HTL, *P. aeruginosa* has receptors LasR and RhlR, which are part of the LuxR protein family, and HTL could compete with the species-specific ligands produced by the bacteria. Our findings link a ubiquitous mechanism for MetRS-catalyzed HTL synthesis to bacteria previously considered silent because they lack homologs of the LuxI synthase, giving them voice. This resolves important questions about bacterial communication and fosters new ideas about the interaction of HTL with canonical QS elements.

## MATERIALS AND METHODS

### Strains and culture media

All experiments in this work were performed using WT or mutants of the parental strain *E. coli* K-12 BW30270. Additional sequencing analysis of the BW30270 strain revealed a previously undescribed mutation in a locus associated with bacterial motility (Fig. S3), which was uniformly distributed across all BW30270 strains used in this study. Strains and plasmids are listed in Table S1. Strains were cultured in LB media (1% tryptone, 0.5% yeast extract, and 0.5% NaCl), M9 media (47.7 mM Na_2_HPO_4_, 22.0 mM KH_2_PO_4_, 8.6 mM NaCl, 18.7 mM NH_4_Cl, 2 mM MgSO_4_, 0.1 mM CaCl_2_, thiamine 1 µg/mL, FeSO_4_·7H_2_O 0.5 µg/mL, and 0.4% glucose). When indicated, amino acids (40 µg/mL each, except Met), Met (320 µM), Hcy (320 µM), HTL (320 µM or 500 µM), N-octanoyl-L-homoserine lactone (C8-HSL; 320 µM), N-(3-oxooctanoyl)-L-homoserine lactone (3OC8-HSL; 320 µM), DL-HTL (320 µM), ampicillin (100 μg/mL), arabinose 0.4%, or glucose 0.4% were added to the culture media.

### Growth curves

*E. coli* overnight cultures in LB media were washed three times with fresh M9 media and diluted to an OD_600nm_ ~ 0.1 in M9. Aliquots of 10 µL were transferred to a 96-well microplate with 190 µL of M9 in each well. Growth was monitored on a Tecan SPARK microplate reader by measuring absorbance at 600 nm at 37°C, with constant shaking, alternating between orbital (142 rpm, 6 mm width) and linear (296 rpm, 6 mm width) shaking. Doubling and lag times were calculated from the slope and *x*-intercept of the exponential growth curve, respectively.

### HTL quantification

*E. coli* cultures were performed following the same protocol as the growth curves but in a volume of 12 mL of M9. At each point along the curve, 1 mL of culture was collected, its OD_600_ measured, then centrifuged for 2 min at 16,000*g* (room temperature), and 800 μL of the supernatant was stored at −80°C until analysis. Aliquots of 100 μL were mixed with 5 μL of 4 M NaOH and incubated for 15 min at 42°C to hydrolyze thiolactone into homocysteine. This solution was cooled to room temperature and then neutralized with 5 μL of 4 M HCl. A total of 100 μL of this solution was transferred to a black 96-well microplate containing 100 μL of 4 mM OPA in PBS and immediately transferred to a plate reader to measure the fluorescence intensity (Ex. 370 ± 20 nm, Em. 420 ± 20 nm) over a period of 30 min every 20 s (26, 28). Triplicate of HTL hydrolysis and OPA reaction were performed for each sample.

### Cloning and mutation protocols

PON1 is a mammalian serum paraoxonase whose expression in *E. coli* has been limited due to its aggregation and lack of glycosylation. In this study, we used the G3C9 variant, a rabbit PON1 developed to improve its expression in *E. coli* (29). PON1-G3C9 was amplified from plasmid pG3C9 using primers EcoRI-PON1 and KpnI-PON1 and cloned into the low-copy, arabinose-inducible plasmid pBAD30 using restriction enzymes EcoRI and KpnI. sdiA was amplified from genomic DNA of *E. coli* BW30270 using primers EcoRI-SdiA-fw and HindIII-SdiA-H8-rv (Table S2), which added an 8HisTag at the N-terminus (pBAD-SdiA-H8), and cloned into plasmid pBAD30. *metG*∆*sdiA* was constructed by the Red-swap method (63) using primers sdiA(H1 + P1) and sdiA(H2 + P2) (Table S2) and plasmid pKD4 as a template for amplification of a KanR resistance cassette flanked by FRT sites. BW30270 was transformed with pKD46 to catalyze recombination. All constructs were verified by Sanger sequencing.

### RNA extractions

*E. coli* ON cultures in LB medium were washed three times with fresh M9 medium and incubated for 1 h at 37°C with shaking to adjust to the media change. The cultures were split, and half was supplemented with 320 µM HTL. One milliliter of each bacterial culture was pelleted for 1 min at 12,000*g*. The pellet was resuspended in 50 µL of lysis buffer (83 mM Tris-HCl, pH 6.8, 18 mM EDTA, pH 8, 1.7% SDS, and 1.6% 2-mercaptoethanol) and incubated for 3 min at 37°C. A total of 1.5 mL of TRIzol was added, and total RNA was extracted following the manufacturer’s recommended protocol.

### Quantitative reverse transcription polymerase chain reactions

Total RNA (5 µg) was treated with TURBO DNase (Invitrogen), followed by a reverse transcription reaction with 2.4 µg RNA, random primers, and SuperScript IV (Invitrogen) according to the manufacturer’s protocol. The resulting cDNA was used for real-time PCR with primers specific for the *gadY* or *rmf* genes (Table S2) and SsoAdvanced Universal SYBR Green Supermix (Bio-Rad) in a CFX Connect Real-Time PCR Detection System (Bio-Rad). Changes in expression level in M9 or M9 + HTL media were represented as log_2_ fold change (ΔCt_M9_-ΔCt_HTL_) (64).

### Differential scanning fluorimetry

Purified SdiA-H8 (final concentration 3.1 µM) and ligand (final concentration 5 mM) were mixed and incubated for 30 min at 4°C. After the addition of SYPRO Orange fluorophore (Sigma), the reaction was immediately transferred to a precooled CFX Opus 96 real-time PCR system. Cycles of 20 s incubation and fluorescence measurement (fluorescence resonance energy transfer [FRET] scan mode) were performed in a thermal scan from 4°C to 90°C (+0.5°C/cycle). The Tm_*a*_ and maximum fluorescence (Max) were determined from the derivatives of the fluorescence curves, using a protein: fluorophore molar ratio of 1:2.6. In contrast, the fluorophore quenching was determined with a ratio of 1:2.26. The same procedure was applied for Hcy, Met, and ligand vehicle (H_2_O) controls.

## SUPPLEMENTAL MATERIAL

Supplemental material mbio.01407-26-s0001.pdf Additional experimental details; supplemental figures and tables.

ASM does not own the copyrights to Supplemental Material that may be linked to, or accessed through, an article. The authors have granted ASM a non-exclusive, world-wide license to publish the Supplemental Material files. Please contact the corresponding author directly for reuse.
