# Supplementary material for: Homocysteine thiolactone from translation proofreading is an endogenous ligand for cell-cell signaling by the receptor SdiA
Source: mBio. 2026 Jul 16;17(8):e01407-26. doi: 10.1128/mbio.01407-26 (PMC13463772; doi:10.1128/mbio.01407-26)
Supplement: Supplementary file 1 [file mbio.01407-26-s0001.pdf]

Supplementary Material

SdiA purification

SdiA purification was performed using *E. coli* BL21-DE3/XJ1 (Table S1). One-liter LB cultures supplemented with Amp were inoculated with 5% ON preinoculum and grown for 3 h at 37°C. SdiA-H8 was induced with 10 mM Ara for 3 h under the same growth conditions. Cells were harvested by centrifugation (4000g, 4°C) and pellets were stored at -80°C until use. Bacteria were resuspended in 10 mL of lysis buffer (25 mM sodium phosphate buffer, pH 7.4, 150 mM NaCl, 1% Triton-X100, and protease inhibitor cocktail) and sonicated on ice. The lysate was centrifuged (25,000 g, 30 min, 4°C), and the recovered supernatant was fractionated by protein precipitation between 1.2 and 1.6 M ammonium sulfate. The pellet was resuspended in 10 ml of lysis buffer, filtered (0.45 µm, nitrocellulose), and injected into ÄKTA start FPLC (Cytiva). Due to the charge of SdiA-H8, a cation exchange column (1 mL, HiTrap Q FF, Cytiva) and a nickel column (1 mL, HisTrap FF, Cytiva) were connected in tandem for protein loading. After disconnection of the exchange column, the nickel column was washed with buffer A (25 mM sodium phosphate, pH 7.4, 25 mM imidazole, and 800 mM NaCl), buffer B (25 mM sodium phosphate, pH 7.4, 115 mM imidazole, and 150 mM NaCl), and eluted with buffer E (25 mM sodium phosphate, pH 7.4, 500 mM imidazole, and 150 mM NaCl). SdiA-H8 was dialyzed in 10,000 MWCO dialysis tubing (Slide-A-Lyser®Mini dialysis units, Thermo Scientific) in HEPES Buffer (20mM HEPES, pH 7.4, 100mM NaCl). The purity of SdiA-H8 was analyzed by SDS-PAGE and Western blot anti-Hisx6.

Translation Efficiency Analyses. *E. coli* strains were transformed with the plasmid pMet-*gfp*, which contains an arabinose-inducible operon composed of a superfold green fluorescent protein (sfGFP) variant with 4 Met repeats at the sixth codon, followed by a modified mCherry fluorescent protein (65). Bacteria from an ON culture were inoculated into M9 medium supplemented with amino acids and

ampicillin and grown at 37°C with constant shaking. When bacteria reached the mid-log phase (OD600 ~0.4–0.6), a 50 µl aliquot was transferred to a 96-well optical bottom plate containing 150 µl of fresh M9 medium supplemented with arabinose (final concentration 0.4%) and Met where indicated. The plates were incubated at 37°C with constant shaking in a microplate reader (SPARK, Tecan), where absorbance (600 nm), fluorescence intensity of green fluorescent protein (GFP; Ex. 480 ± 4.5 nm, Em. 515 ± 10 nm) and mCherry (Ex. 555 ± 4.5 nm, Em. 600 ± 10 nm) were periodically recorded.

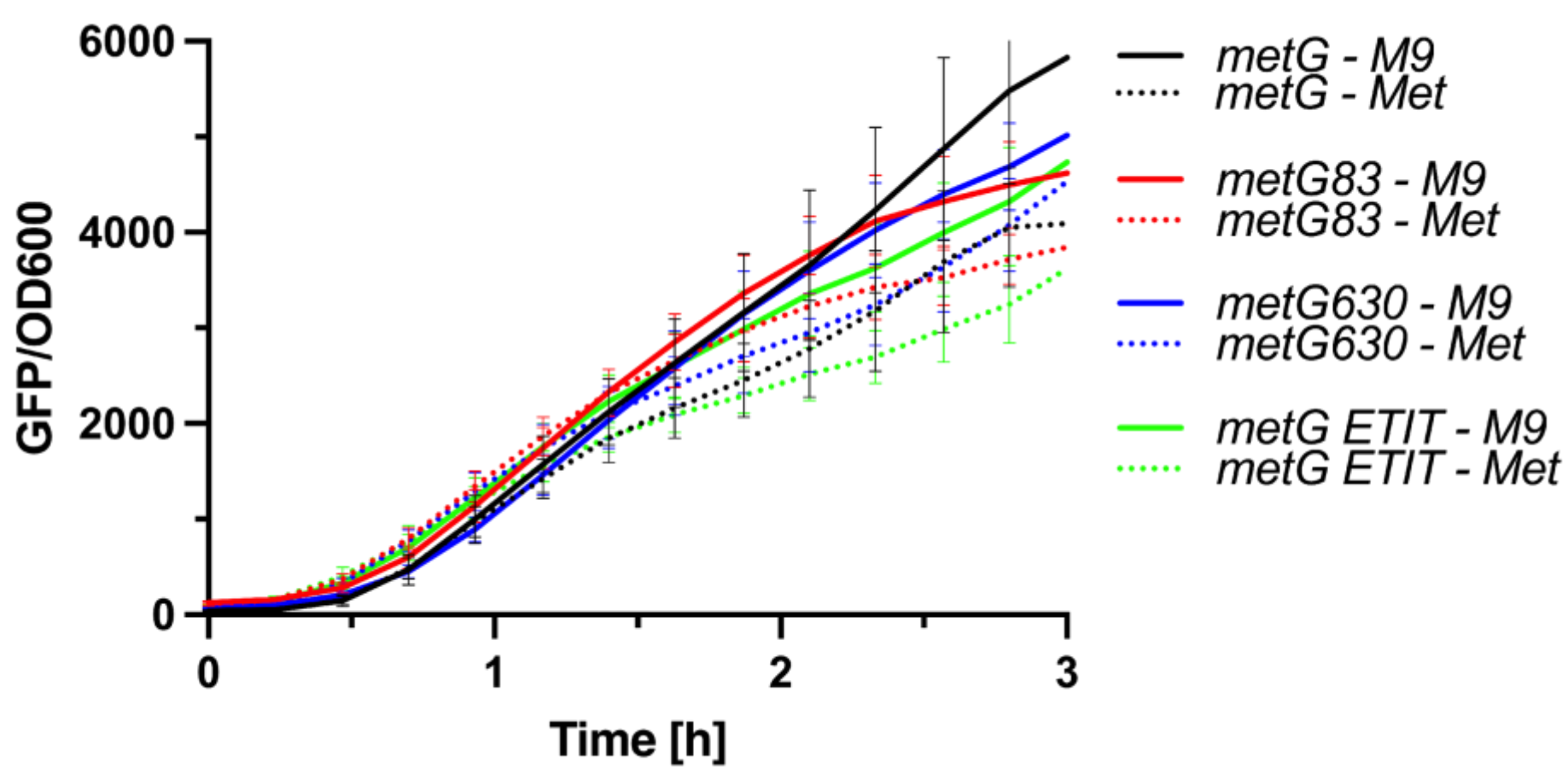

Figure S1. Comparison of protein synthesis between *metG* mutants. Strains *metG* (wild-type), *metG83*, *metG630*, and *metG ETIT* were transformed with a reporter system encoding the GFP superfolder (GFP) downstream of the inducible PBAD promoter. Kinetics of fluorescence intensity normalized by OD600 (GFP/OD600) was performed on M9, where glucose is replaced by glycerol (M9) and medium supplemented with Met (dotted lines).

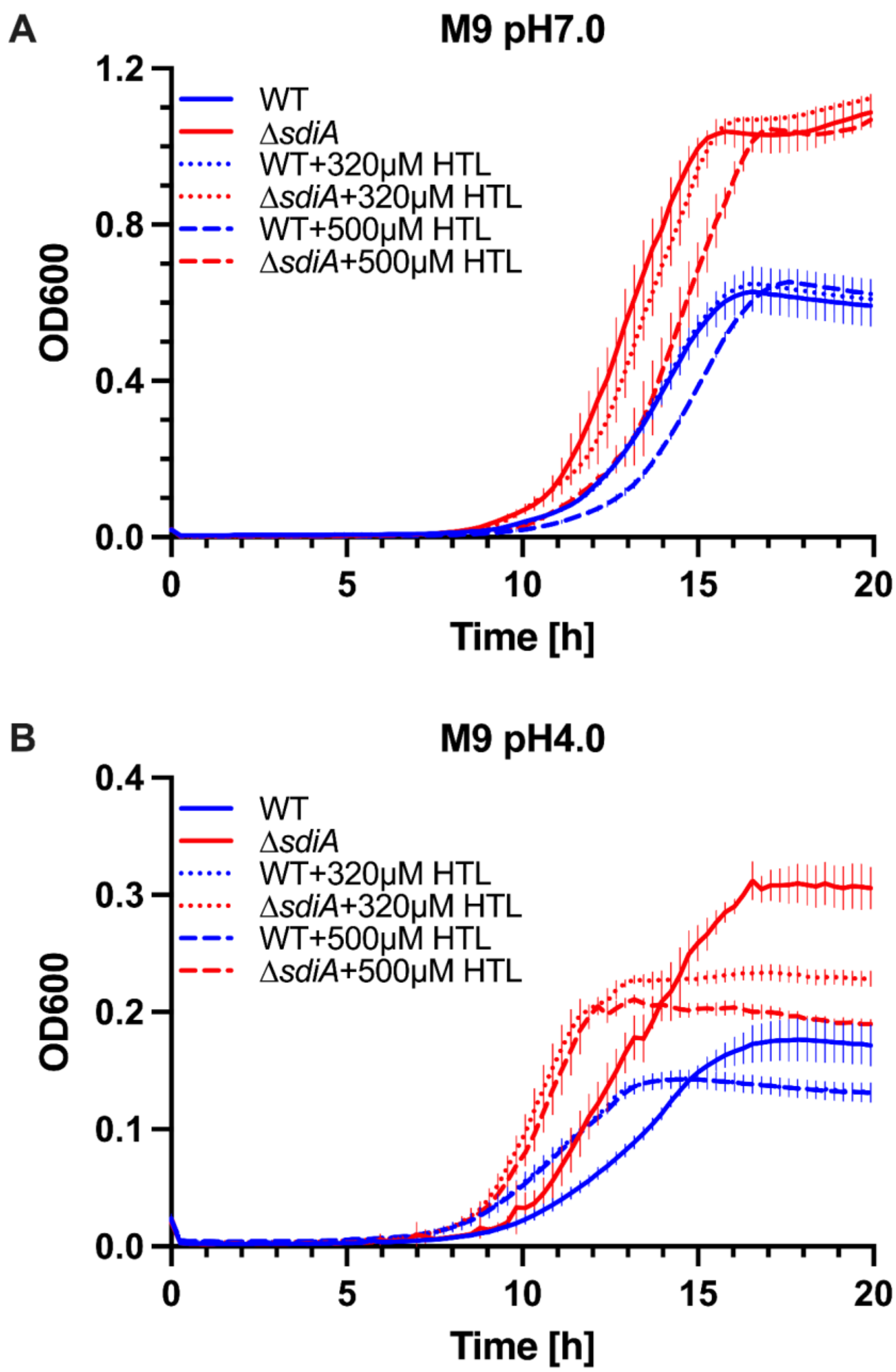

Figure S2. Analysis of the effect of HTL on *E. coli* K12 MG1655. Wild-type (WT) and *sdiA* mutant (Δ*sdiA::FRT*) *E. coli* strains were grown in M9 medium at pH 7.0 (A) and pH 4.0 (B), supplemented with 320 µM (dotted lines) and 500 µM (dashed lines) of HTL, as indicated. Optical density at 600 nm (OD600) was monitored in a microplate reader at 37 °C with constant shaking.

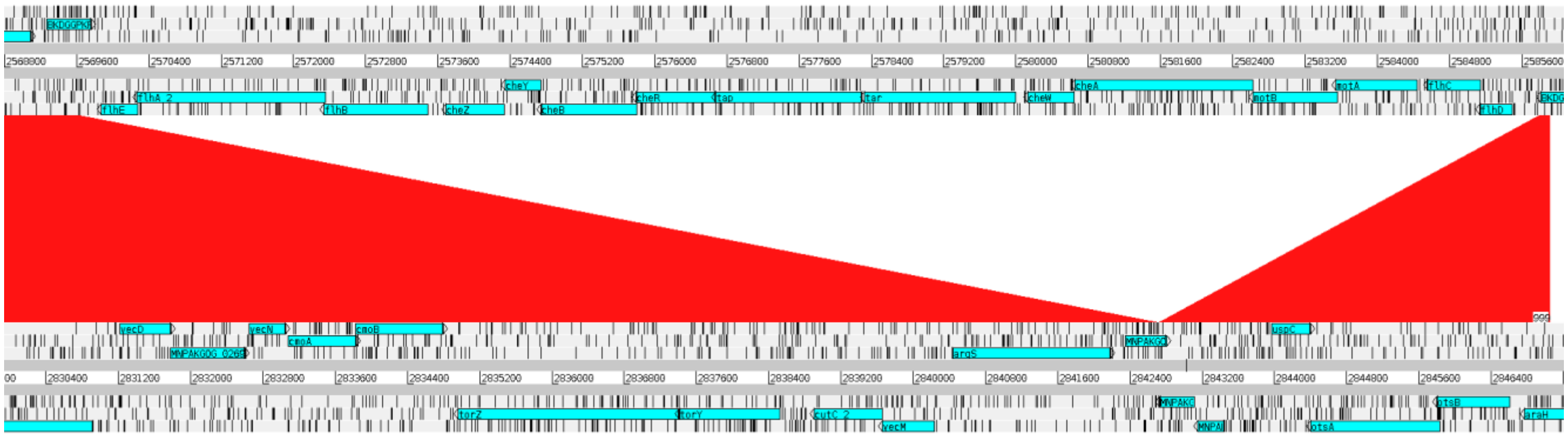

Figure S3. Deletion of a motility locus in *E. coli* K12 BW30270. Alignment of the genomes of strain MG1655 (upper sequence) and BW30270 (lower sequence). The red color indicates the match between the genomes, delimiting an approximately 16 kb deletion that includes the genes *flhD, flhC, motA, motB, cheA, cheW, tar, tap, cheR, cheB, cheY, cheZ, flhB, flhA, and flhE*. The sequences were aligned using NCBI-BLAST and visualized with the Artemis Comparison Tool (ACT).

Molecular docking analysis

To compare the interaction between SdiA and potential ligands, a molecular docking analysis was performed using the Cavity-detection-guided Blind Docking 2 (CB-Dock2) server (66). This server detected cavities in the 3D structure of SdiA (AF-P07026-F1-model-v4) for a subsequent molecular docking step using the AutoDock Vina docking engine. The cavity selection was based on the structure of the SdiA (fragment 1-171)/C8-HSL complex (5) (Figure S4). Molecular docking was performed using the structures of the molecules L-HTL (ChEBI ID: 60315), C4-HSL (ChEBI ID: 29643), C6-HSL (ChEBI ID: 181934), 3OC6-HSL (ChEBI ID: 63789), C8-HSL (ChEBI ID: 195581), 3OC8-HSL (ChEBI ID: 181091), C10-HSL (ChEBI ID: 190815), and C12-HSL (ChEBI ID: 220910). Then, the software calculated the Vina score, which corresponds to the binding energy ($\Delta G_{docking}$) of the interaction. Finally, the binding affinity was normalized by the size of the molecules by dividing the additive inverse of the Vina score by the number of heavy (non-hydrogen) atoms (N) in the ligand (-Vina score/N) to obtain the Ligand Efficiency.

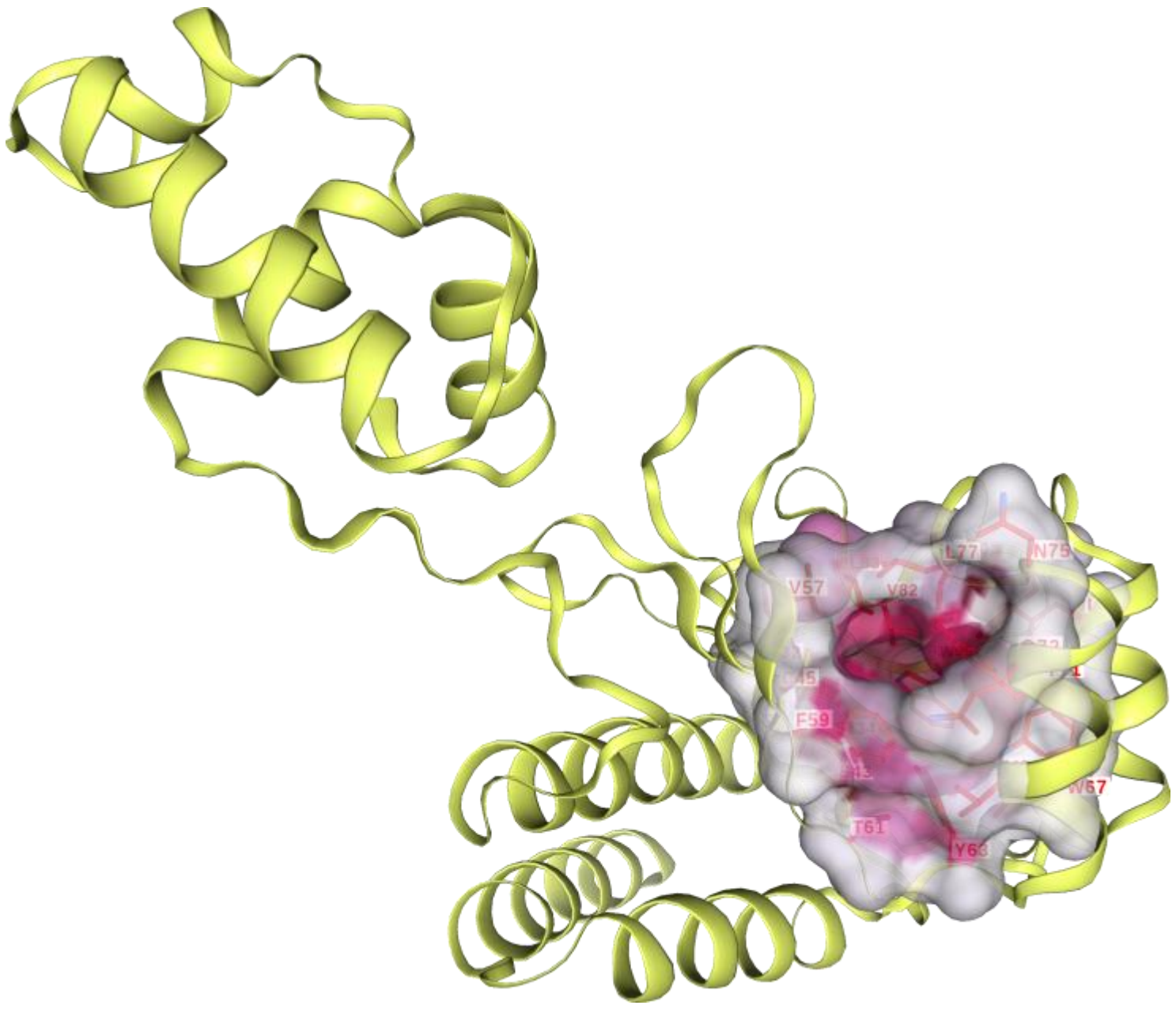

Figure S4. SdiA binding pocket analysis. Based on the 3D structure AF-P07026-F1-model-v4 of SdiA (in yellow), the CB-Dock2 server identified the binding cavity (surface in gray) and the amino acids involved in the interaction with L-HTL. The cavity has a volume of 616 Å³, and the amino acids involved in the interaction are the same as those identified by NMR (Figure 3D).

Table S1. Escherichia coli strains and plasmids used in this study.

Strain or plasmid	Relevant Features	Source or Reference
Strains		
metG	BW30270 (wild-type strain); MG1655 rph+, Δ[*flhD, flhC, motA, motB, cheA, cheW, tar, tap, cheR, cheB, cheY, cheZ, flhB, flhA, flhE*]	Gregory J. Phillips Laboratory
metG 630	BW30270; *metG mutant* (-4TGAT2183)	Wood et al., 2024
metG 83	BW30270; *metG mutant* (G263S, S264F)	Wood et al., 2024
metG ETIT	BW30270; *metG mutant* (ΔGAAACCATCACC2011)	Wood et al., 2024
metGΔsdiA	BW30270 Δ*sdiA::KanR*	This study
Plasmids		
pG3C9	pET32b(+)-$PON1_{G3C9}$	Aharoni et al., 2004
pPON1	pBAD30-$PON1_{G3C9}$	This study
pKD4	Ori_{R6K}, KanR	Datsenko and Wanner, 2000
pKD46	oriR101 w/repA101ts, AmpR	Datsenko and Wanner, 2000
pMet-*gfp*	PBAD30SFIT-sfgfp(4xMet), mCherry	Leiva et al., 2022

Table S2. Primers used in this study.

Oligonucleotide	Sequence (5'→3')
EcoRI-PON1	CCG*gaattc*AGGAGGAATTTACCATGGCTAAACTGACAGCGC
KpnI-PON1	CGG*ggtacc*TAACAGCTCACAGTAAAGAGC
EcoRI-SdiA-fw	CCG*gaattc*AGGAGGAATTTACCATGCAGGATAAGGATTTTTC
HindIII-SdiA-H8-rv	CCC*aagctt*TCAGTGATGGTGATGGTGATGGTGATGTTCATCATTTAAACGCATCAG
sdiA(H1+P1)	TTATAAATGATACTCACTCTCAGGGGCGTTGCGGTTTACTATGCAGGATgtgcaggctggagctgcttc
sdiA(H2+P2)	CAGCCGGTTTTTGCATCTGGCACGCAGGACAGAAAAGAGATCAAATTAAcatatgaatatcctccttag
*gad*Y-fw	CACAAAGTTTCCCGTGCCAA
*gad*Y-rv	GGGACCGGGAAGAGGATAGT
rmf-fw	GCCGGACGCTCAAAAGAAAT
rmf-rv	GGCCATTACTACCCTGTCCG

* Lowercase Italic letters indicate restriction sites.

** Lowercase underlining letters indicate the region that binds to the antibiotic resistance cassette used for mutagenesis.

References

65. Leiva LE, Elgamal S, Leidel SA, Orellana O, Ibba M, Katz A. 2022. Oxidative stress strongly restricts the effect of codon choice on the efficiency of protein synthesis in Escherichia coli. Front Microbiol 13:1042675. https:// doi.org/10.3389/fmicb.2022.1042675

66. Liu Y, Yang X, Gan J, Chen S, Xiao Z-X, Cao Y. 2022. CB-Dock2: improved protein–ligand blind docking by integrating cavity detection, docking and homologous template fitting. Nucleic Acids Res 50: W159–W164. https://doi.org/10.1093/nar/gkac394
